# Primary subtalar arthrodesis in displaced intra-articular calcaneal fracture: a systematic review

**DOI:** 10.1007/s12306-025-00901-0

**Published:** 2025-05-15

**Authors:** A. Giuliani, S. Calori, A. Singlitico, F. Forconi, G. Maccauro, R. Vitiello

**Affiliations:** 1 Department of Ageing, Neurosciences, Head-Neck and Orthopedics Sciences, Orthopedics and Trauma Surgery Unit, Fondazione Policlinico Universitario Agostino Gemelli IRCCS, Largo Francesco Vito 1, 00168 Rome, Italy; 2 Orthopedics and Trauma Surgery, Università Cattolica del Sacro Cuore, Largo Francesco Vito 1, 00168 Rome, Italy; 3 UniCamillus-Saint Camillus International University of Health Sciences, 00100 Rome, Italy; 4 Casa di Cura Villa Stuart, Via Trionfale, 5952, 00136 Rome, Italy

**Keywords:** Calcaneal, Fracture, Subtalar, Arthrodesis

## Abstract

Calcaneus fractures are severe injuries often resulting from traumatic falls or motor vehicle accidents. Surgical treatment through open reduction and internal fixation (ORIF) is considered the standard approach for displaced intra-articular calcaneal fractures (DIACFs), but it is associated with many complications. Our study aimed to review the current literature available on primary subtalar arthrodesis (PSA) as a first-line treatment for DIACFs, mostly Sanders type IV. In this study, we conducted a systematic review following the Preferred Reporting Items for Systematic Reviews and Meta-Analysis (PRISMA) guidelines. The keywords were searched in PubMed, MEDLINE and the Cochrane Library. This review included articles where primary arthrodesis was performed in calcaneal fractures, with or without associated implants. Nine articles were included in the review. The total population comprised 184 patients with 192 calcaneal fractures. The mean age was 44.9 ± 6.9 years old. The mean follow-up period was 30.28 ± 15.29 months when reported. The mean time to surgery was 13.33 ± 7.02 days from injury. All studies reported a good fusion rate (between 94 and 100%) and an average fusion time of 4.05 ± 2.19 months. The mean American Orthopedic Foot & Ankle Society (AOFAS) score was 71.26 ± 8, and the mean Visual Analog Scale (VAS) score for pain was 3.26 ± 0.91. Primary arthrodesis of the subtalar joint for treating DIACFs, mostly Sanders type IV, provides good results due to the avoidance of further procedures, reduced postoperative pain, and a high rate of bony union. However, success heavily depends on factors such as patient comorbidities and addressing hindfoot deformity. Further studies with larger patient populations and more standardized protocols are necessary to draw definitive conclusions about the best management strategies for DIACFs. Systematic review, level III of evidence.

## Introduction

Calcaneus fractures are serious injuries, often resulting from traumatic falls or motor vehicle accidents [1]. These fractures represent the most common type of tarsal bone fracture, accounting for up to 2% of all fractures in the human body, with 75% of these fractures being intra-articular [2].

Clinically, the injuries manifest with swelling, pain, distal edema at the level of the calcaneus cuboid joint, and ecchymosis. A significant clinical consequence of this injury is the inability to ambulate. Sometimes, a deformity of the anatomical profile of the hindfoot is noticeable, while vasculo-nervous lesions are rare [3].

Radiographic classification of calcaneal fractures is essential for guiding treatment. The Essex-Lopresti classification, one of the earliest referenced systems, categorizes fractures into tongue-type and joint depression-type based on the fracture pattern and direction of the primary fracture line [4]. This classification remains relevant today, particularly for determining surgical approaches and predicting complications.

Additionally, the Sanders system, currently one of the most widely utilized classification systems for calcaneal fractures, categorizes intra-articular fractures into four types (Type I, II, III, and IV) based on the number and location of fracture lines and fragments [5, 6].

Sanders and colleagues described calcaneal fractures as follows: Type 1, nondisplaced; Type 2, displaced with the posterior facet in two fragments (with A, B, and C to denote fracture location); Type 3, with the posterior facet having three major fracture fragments; and Type 4, comminuted. The prognostic implications of the Sanders classification are critical in determining the appropriate treatment strategy [5, 7].

Historically, displaced intra-articular calcaneal fractures (DIACFs) were treated nonoperatively, as predictable operative reduction and fixation were not possible [8]. Presently, surgical treatment through ORIF is considered the standard approach for DIACFs [9].

Although ORIF has improved clinical and radiological outcomes of DIACFs, Sanders and colleagues reported that fractures involving four or more parts of the posterior facet of the subtalar joint result in poor outcomes and complications after surgery, proposing primary subtalar arthrodesis (PSA) as alternative [10–13].

Several authors advocate PSA in cases of highly comminuted fractures due to favorable functional results [11, 13, 17]. The management of these fracture patterns is not well established, and the literature remains controversial.

The aim of our study was to review the current literature on PSA as the first-line treatment in DIACFs, mostly Sanders type IV, to assess clinical and radiological outcomes and potential complications.

## Materials and methods

**Fig. 1:**
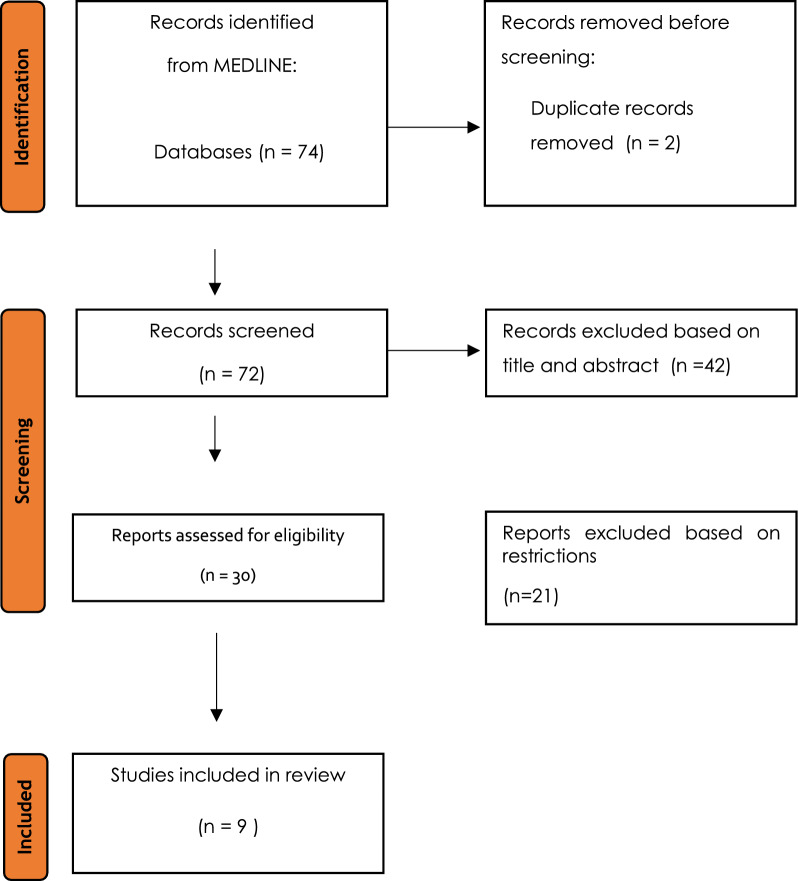
PRISMA flowchart [14]

In this study, a systematic review was performed according to the Preferred Reporting Items for Systematic Reviews and Meta-Analysis (PRISMA) guidelines (Fig. 1) [15].

The keywords “calcaneal”, “calcaneus”, “fracture”, “arthrodesis”, “fusion” and their MeSH terms were searched in PubMed, MEDLINE and Cochrane Library.

To minimize the number of missed studies, no filters were applied to the search strategy. No restrictions on the date of publication were applied; the only restriction applied was the language (only articles in English). In this review, we included articles where PSA was performed in calcaneal fractures, with or without associated implants.

**Fig. 2:**
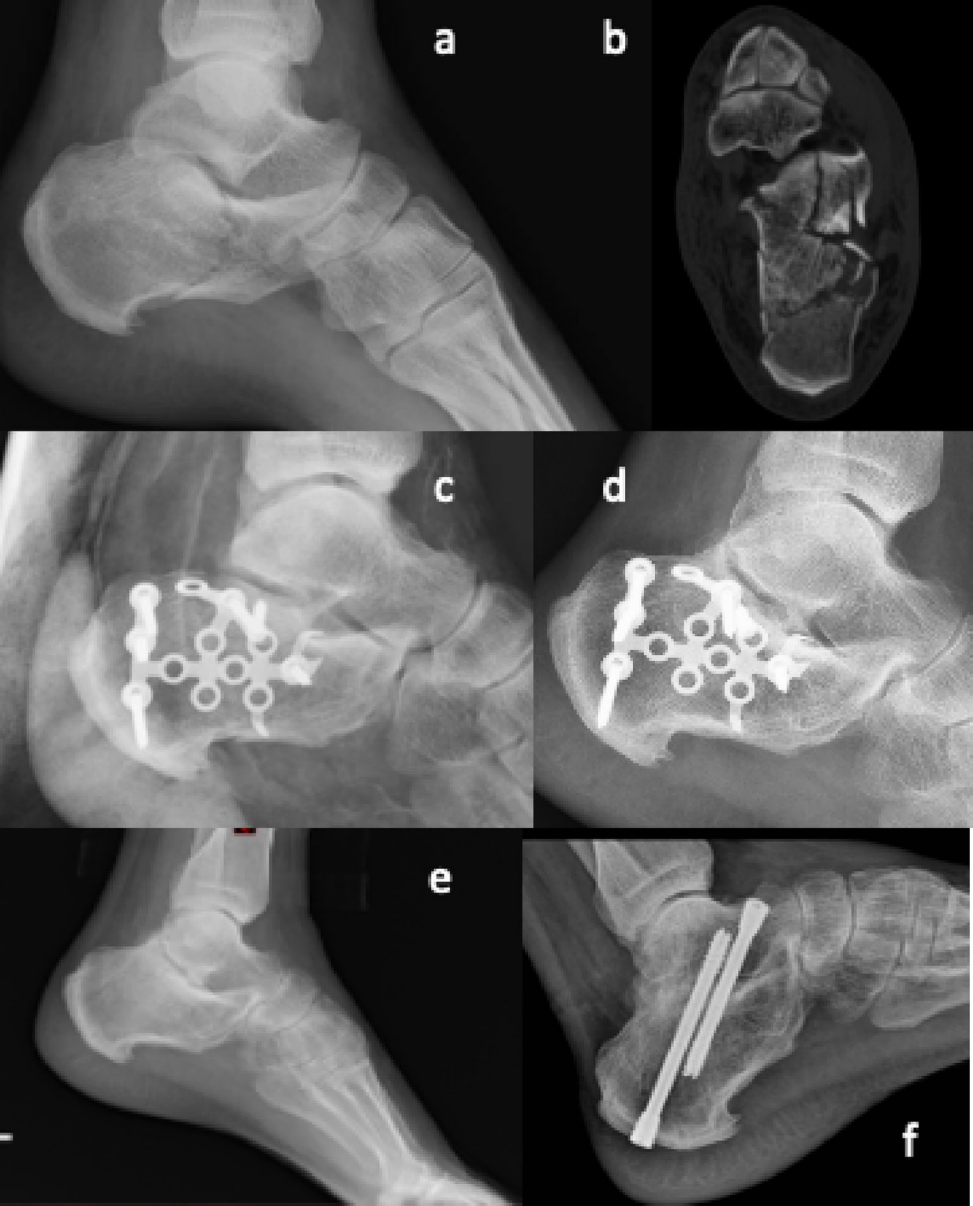
Clinical case of calcaneal fracture about our Institute (Department of Ageing, Neurosciences, Head-Neck and Orthopedics Sciences, Orthopedics and Trauma Surgery Unit, Fondazione Policlinico Universitario Agostino Gemelli IRCCS). **a** Lateral X-ray view of calcaneal fracture; **b** preoperative axial view of CT scan; **c** postoperative lateral view of ORIF for calcaneal fracture; **d** 1-month postoperative X-ray control; **e** secondary subtalar arthritis 3 years after surgical treatment; **f** subtalar arthrodesis made by two cannulated partially treated screws, postoperative X-ray control, 5 years after first treatment

Two independent authors (S.C and A.S) screened abstracts and full texts; any discordance was resolved by consensus of a third author. The methodological quality of the studies was assessed using the modified Coleman Methodology Score (mCMS) [14], which ranges from 0 to 100 points, representing a well-designed study with no bias or confounding factors (Fig. 2).

The following data were extracted: demographic data; clinical outcomes through physical examination and rating scales, primarily AOFAS and VAS; radiological outcomes such as time of bone union and measurements of pre- and postoperative tarsal-metatarsal angles; types of implants used; short- and long-term complications reported by the authors.

## Results

The electronic search resulted in 74 articles. Following the PRISMA flowchart [15], only nine studies met the inclusion criteria and were included in the systematic review [16–24]. Other papers were excluded for the following reasons: non-English written paper, systematic reviews, and studies involving secondary arthrodesis. According to the mCMS evaluation, the mean score of the studies was 33.4 ± 7.28. This review included 184 patients, totaling 192 calcaneal fractures treated by PSA. Two studies compared PSA results with ORIF results, but in our analysis, we only considered the PSA results [18, 19]. Neumaier et al. evaluated the results of DIACFs treated by two-step surgery: staged external fixation to combined ORIF and PSA [22]. Other studies examined PSA results, with or without internal fixation, as definitive treatment of DIACFs [16, 17, 20, 21, 23, 24].

### Demographic and surgical data

We reached a population of 184 male and female patients with a total of 196 calcaneal fracture, the vast majority of which were closed fractures (183 cases), and 13 were open fractures. The number of male and female patients was 115 and 34, respectively. The mean age was 44.9 ± 6.9 years. All the studies reported the preoperative Sanders classification of DIACFs except one [16]. Two studies included open fracture [21, 22]. The mean follow-up period was 30.28 ± 15.29 months when reported. The mean time to surgery was 13.33 ± 7.02 days from injury. Demographic results are reported in Table 1.

**Table 1:** Demographic data

References	Number of patients; injuries	Sanders classification	Gender [Male (M); female (F)]	Age (mean: year)	Time to surgery (mean: days)	Follow-up (mean: months)	mCMS
Holm et al. [19]	17; 17	17 Type IV	9 M; 8 F	54.8	14.8	34.4	28
Lopez-Oliva et al. [20]	33; 37	37 Type IV	33 M; 0 F	42.08	6	24	43
Potenza et al. [22]	6; 7	7 Type IV	4 M; 2 F	40	20	32.5	34
Buckley et al. [17]	14; 14	14 Type IV	13 M; 1 F	44	–	54	46
Buch et al. [16]	14; 14	14 Type IV	12 M; 2 F	40	14	26	27
Neumaier et al. [21]	12; 13 (1 bilateral fracture)	9 Type IV 4 Type III	10 M; 2 F	41.9	31.1	13	34
Schipper et al. [23]	35; 35	1 Type I 14 Type III 20 Type IV	–	47.8	–	34.4	40
Agren et al. [15]	29; 35 (6 bilateral fractures)	–	20 M; 9 F	37	–	–	15
Dingemans et al. [18]	24; 24	11 Type II 8 Type III 5 Type IV	14 M; 10 F	57	–	–	34
Mean value ± STDEV	–	–	14.35 ± 8.79 M; 4.25 ± 4.02 F	43.06 ± 2.3	13.33 ± 7.02	30.28 ± 15.29	33.44 ± 7.28

mCMS, modified Coleman methodology score; STEDV, standard deviation

Concerning surgical information, most of the included studies described one-step surgery for the definitive treatment of DIACFs trough PSA, with or without ORIF, using one, two or more cannulated screws (partially or fully threated) [16, 17, 20–23].

Holm et al. showed results of PSA using large fragment screws in 16 patients and Kirschner wires and an external fixator in one patient, affected by severe obesity [20].

Lopez et al. focused on results regarding a particular subtalar fusion navigated system made with two cannulated screws (Vira®System) [21].

Neumaier et al. described their surgical approach in two stages for 13 calcaneal fractures, ten of which were open fracture. In the first stage, they performed wound irrigation and external fixation. In the second stage, they removed the external fixator and performed ORIF plus PSA, using large fragment cannulated screws, partially or fully treated, for arthrodesis, and a lateral calcaneal plate for internal fixation. For more comminuted variants, fully threaded screws or long thread partially threaded screws was used for subtalar arthrodesis to avoid collapse through the comminution [22].

The use of bone graft was reported in four studies [17, 18, 20, 22] where cancellous or autogenous bone grafts were used in patients who reported severe bone loss, severe damage of posterior facet and severe chondral loss. Surgical information are reported in Table 2.

**Table 2:** Surgical data

References	Number of patients; fractures	Type of fixation for PSA	Combined ORIF (n° of patients)	Bone graft
Holm et al. [19]	17; 17	16: Large Fragment Screws 1: Kirschner Wires and External Fixator	2	Cancellous chips or autograft bone
Lopez-Oliva et al. [20]	33; 37	Two navigated cannulated screws (Vira®System)	–	–
Potenza et al. [22]	6; 7	Two/three partially treated 6.5-mm cannulated screws	–	–
Buckley et al. [17]	14; 14	Partially and fully treated 7.3-mm cannulated screws	–	Autograft (ipsilateral iliac crest)
Buch et al. [16]	14; 14	One 7-mm fully treated cannulated screw	14	Autograft bone (iliac crest) or cancellous bone
Neumaier et al. [21]	12; 13 (1 bilateral fractures)	Cannulated (partially/fully treated) screws	13	Cancellous bone from tuberosity
Schipper et al. [23]	35; 35	Cannulated screws	–	–
Agren et al. [15]	29; 35 (6 bilateral fractures)	–	–	–
Dingemans et al. [18]	24; 24	–	–	–

### Radiological and clinical data

The studies included in our review focus on radiological and clinical results after PSA. Radiological data, when reported, were measured by the angles on pre- and postoperative x-rays or CT scan, while clinical data were collected through physical examination and rating scales.

The average talo-calcaneal (TC) angle reported by Buch et al. was 20.6 degrees, indicating postoperative varus deformity of hindfoot; it is relevant to note that nine patients showed radiographic evidence of some degree of calcaneocuboid arthritis. Two other patients had evidence of spontaneous calcaneocuboid fusion without arthritis [17]. Two studies reported physiological restoration of the talus-first metatarsal angle after PSA [17, 20].

All studies showed a good fusion rate (between 94 and 100%) with an average fusion time of 4.05 ± 2.19 months, when reported. Radiological results are reported in Table 3.

**Table 3:** Radiological results

References	Bohler’s angle (mean) (°)	Talo-first metatarsal angle (mean) (°)	Talo-calcaneal angle (mean) (°)	Time to radiographic fusion (months) or % at the end of follow-up
Holm et al. [19]	28.1	1.9	36.7	100%
Lopez-Oliva et al. [20]	–	–	–	–
Potenza et al. [22]	–	–	–	100%
Buckley et al. [17]	–	–	–	–
Buch et al. [16]	–	7.6	20.6	2.5
Neumaier et al. [21]	–	–	–	5.6
Schipper et al. [23]	26.3	–	28.6	94.3%
Agren et al. [15]	–	–	–	–
Dingemans et al. [18]	–	–	–	–
Mean value ± STDEV	27.2 ± 1.27	4.75 ± 4.03	28.6 ± 8.05	4.05 ± 2.19

FU, follow-up

Regarding clinical findings, all studies demonstrated good or mildly good postoperative results, as reported in Table 4. Notably, in the study of Neumaier et al*.,* the primary patient-reported outcome measure was PROMIS data, divided into depression, pain interference and physical function, with mean scores of 52.1, 62.2, and 37.4, respectively. This was one of the first studies to use PROMIS in the context of calcaneus fractures [22].

**Table 4:** Clinical results

References	Weeks to full weight bearing	AOFAS-AHS (mean score)	SF-36 (mean score)	VAS (mean score)
Holm et al. [19]	8	78.4	–	1.9
Lopez-Oliva et al. [20]	–	75.43	–	–
Potenza et al. [22]	10	80	–	–
Buckley et al. [17]	6	65.75	33.03	3.71
Buch et al. [16]	8.6	72.4	–	–
Neumaier et al. [21]	10	–	–	3.6
Schipper et al. [23]	8	–	–	–
Agren et al. [15]	–	56.9	56.2	3.86
Dingemans et al. [18]	–	70.0	52.3	–
Mean Value ± STDEV	8.4 ± 1.67	71.26 ± 8	47.17 ± 12.4	3.26 ± 0.91

AOFAS-AHS, American Orthopedic Foot and Ankle Society-Ankle Hindfoot scale; VAS, visual analog scale; SF-36, short form health survey 36

Regarding complications, the most reported issues were wound problems, mostly managed over time with local wound care. Lopez et al*.* reported a deep infection in one patient, with an open fracture, which was treated by implant removal and antibiotics [21].

Buch et al. described one patient who required a split-thickness skin graft (10 × 12 mm) to cover the wound [17]. Neumaier et al. reported three patients with deep infection who required surgical irrigation and debridement of the wound [22].

Complications are reported in Table 5.

**Table 5:** Complications

References	Wound complications	Bone/implants infection	Implants complications	Musculoskeletal problems	Secondary osteoarthritis	Other complications
Holm et al. [19]	–	–	–	–		
Lopez-Oliva et al. [20]	3	1	–	–		2
Potenza et al. [22]	–	–	–	3	2	1
Buckley et al. [17]	–	–	–	–	–	1
Buch et al. [16]	4	–	9	3	9	9
Neumaier et al. [21]	4	–	–	1	–	2
Schipper et al. [23]	–	1	3	–	–	3
Agren et al. [15]						12
Dingemans et al. [18]	3					
Mean value ± STDEV	3.5 ± 0.57	–	6 ± 4.24	2.33 ± 1.15	5.5 ± 4.94	4.28 ± 4.38

## Discussion

This review study reports on the results of nine studies [16–24] that investigated the treatment of DIACFs, mostly Sanders type IV (123 out of 196 fractures), using subtalar arthrodesis with or without ORIF.

Sanders Type IV calcaneal fracture treated by ORIF generally resulted in fair or poor outcomes. This poorer prognosis, which worsens with increasing comminution, has been confirmed by several large studies [25–27].

Literature describes a wide range of complications following ORIF as the definitive treatment of DIACFs [30, 31]. Yu et al. [32] analyzed 21 clinical studies reporting the incidence rates of complications following surgery, identifying infection and skin flap necrosis as the most common complications, with an incidence rate of 13.6%. This rate varies from those reported elsewhere: Tennent et al. [37], and Geel and Flemister [38] reported infection rates of 0.3% and 22%, respectively. These differences may be due to variations in the original injury, unfamiliarity with the anatomy, or inappropriate perioperative management. Post-traumatic osteoarthritis mostly occurs in subtalar and calcaneocuboid joint [39], while malunion or non-union primarily results from mal-reduction and implant problems after ORIF [40]. Other infrequent issues include iatrogenic injuries, such as neurovascular damage [32].

Numerous studies have suggested that DIACFs with severe depression of Böhler’s angle and a high degree of comminution will inevitably require secondary subtalar arthrodesis [12, 33, 34].

Buckley et al. [8] also showed that more severely injured calcaneal fractures (Sanders type III or IV) were at a higher risk of secondary fusions.

The occurrence of post-traumatic subtalar arthritis varies greatly in literature from 2.5% in Poeze’s systematic review to 100% in the long-term (10-year) follow-up by Makki et al. [35, 36]. This wide range is partially due to dissimilar follow-up times and the variety of criteria applied by different authors (radiological signs, symptoms, or need for fusion).

Sanders et al*.* [9] reported that 23% of type III fractures (7 out of 30) and 70% of type IV fractures (8 out of 11) required secondary subtalar arthrodesis. However, with longer follow-up, even less comminuted fractures are at significant risk for secondary arthritis and subsequent fusion (20% in Sanders type 2 and 50% in Sanders type 3 fractures).

Sanders et al*.* concluded that type IV calcaneal fractures are so severe that even the most experienced surgeon may find it difficult to piece these fragments together. Knowing this in advance allows the surgeon and patient to prepare for the possibility of primary fusion [11].

In this review, several studies [17, 20–24] that performed PSA for the treatment of Sanders type IV displaced calcaneal fractures reported good or mildly good clinical and radiological outcomes even 5 years postoperation. Furthermore, a preliminary ORIF to restore the normal height of the calcaneus before performing the subtalar arthrodesis does not seem indispensable to obtain good clinical results; however, this assertion is based on observations reported by the authors of these studies rather than statistical analysis. All these authors described surgical procedure of PSA using two or three partially or fully threated cannulated screws. Buch et al. [17] used only one 7-mm fully threated cannulated screw to avoid later collapse of the hindfoot height.

Agren et al. [16] reported that PSA without considering the deformity of the hindfoot after a calcaneal fracture is not an adequate treatment. They found that the degree of deformity influenced outcome parameters, where patients with the highest degree of deformity had significantly worse VAS score, AOFAS score and SF-36 physical score compared to patients with less deformity. They did not report some demographic and surgical data, such as time to surgery, mean follow-up and type of implants used for PSA.

Authors who compared results between ORIF and ORIF combined with PSA as definitive treatments for DIACFs [18, 19] did not demonstrate any statistical differences between the two treatment options. Buckley et al*.* [20] excluded patients who continued to smoke and those with concomitant injuries, and there was no statistical significance due to the small number of patients. Notably, there is no mention of a prior power analysis to determine the adequacy of the sample size. The authors acknowledged that the limited number of participants could have affected the statistical robustness of their findings, and this represents a significant limitation of the study.

Neumaier et al*.* [22] showed that staged treatment of open or severely comminuted calcaneus fractures with early external fixation and conversion, in a mean of 31.1 days, to combined ORIF and subtalar arthrodesis, using partially or fully threated cannulated screws is a successful treatment protocol with positive improvements in pain and physical function postoperatively. The limitations of this study are the sample size and retrospective nature. It remains to be clarified whether combining the surgical time of calcaneus reduction and fixation with PSA improves clinical and functional outcomes in the follow-up.

Current evidence suggests that when considering surgical options for a DIACFs, many patient factors must be considered to evaluate the correct surgical treatment: comorbidities, age, gender, occupation, etc. [7, 9, 28, 29]

According to current literature and the results we reported, subtalar arthrodesis should be fully considered as a primary and definitive treatment of DIACFs, especially for Sanders type IV.

### Limitations of the studies

The studies included in this review have several limitations that must be acknowledged. First, the sample sizes of most studies were relatively small, which may have impacted the statistical power of their findings. Small sample sizes can increase the likelihood of Type II errors, potentially leading to underestimation of significant differences between treatment approaches. Additionally, there was considerable heterogeneity in the surgical protocols, including variations in the timing of surgery, the type of implants used, and the surgical techniques employed. This lack of standardization makes it challenging to compare results across studies and limits the ability to draw definitive conclusions about the optimal treatment for DIACFs.

Another significant limitation is the lack of standardized follow-up protocols. The follow-up periods varied considerably between studies, and in many cases, the criteria for assessing outcomes were not clearly defined. Some studies relied solely on radiographic outcomes, while others incorporated functional scores and patient-reported outcomes, leading to inconsistent reporting. This variability in follow-up criteria further complicates the interpretation of the results and the generalizability of the findings to broader patient populations.

These limitations highlight the need for more robust, prospective studies with larger sample sizes and standardized protocols to better understand the long-term outcomes of different surgical approaches for DIACFs. Future research should aim to address these gaps to provide clearer guidance on the most effective treatment strategies for these complex fractures.

## Conclusion

Displaced intra-articular calcaneal fractures still pose a challenge to the orthopedic surgeons because they are often associated with poor clinical outcomes and reduced health-related quality of life. The optimal management of these fractures is even more difficult given the lack of consensus on the best approach across different clinical backgrounds.

Our study reported that primary arthrodesis of the subtalar joint for treating DIACFs, mostly Sanders type IV, provides good pain relief, functional outcomes, and radiological results considering the severe nature of the injury.

Furthermore, according to current literature, treating Sanders type IV calcaneal fractures with PSA yields good results due to the avoidance of further procedures, reduced postoperative pain and a high rate of bony union.

Based on the data extracted in this review, PSA represents a valid treatment option for intra-articular calcaneal fractures. However, the success of this approach heavily depends on other critical parameters, such as considering the deformity of the hindfoot after a calcaneal fracture. As discussed, PSA without addressing the hindfoot deformity may not yield adequate outcomes, highlighting the importance of a comprehensive patient evaluation.

Studies with a larger number of patients and more consideration of patient comorbidities are needed, given the small number of randomized studies comparing surgical techniques for managing DIACFs.
